# Multiple emergency department encounters for acute musculoskeletal presentation with an existing mental health diagnosis

**DOI:** 10.1002/ccr3.8010

**Published:** 2023-10-26

**Authors:** Priya Arora, James M Elliott, Fereshteh Pourkazemi, Roxanna Nasseri Pebdani

**Affiliations:** ^1^ Northern Sydney (Arabanoo) Precinct Sydney School of Health Sciences, Faculty of Medicine and Health, The University of Sydney Camperdown New South Wales Australia; ^2^ Northern Beaches Community Mental Health Services (NBCMHS) Brookvale Community Health Centre Brookvale New South Wales Australia; ^3^ Royal North Shore Hospital—The Kolling Institute St Leonards New South Wales Australia; ^4^ Central Sydney (Patyegarang) Precinct Sydney School of Health Sciences, Faculty of Medicine and Health, The University of Sydney Camperdown NSW Australia

**Keywords:** acute pain, emergency presentations, mental disorder, mental illness

## Abstract

Reconceptualising acute Musculoskeletal (MSK) injuries with both stress‐ and tissue‐ based factors is required to consider prior influences of mental health disorders on acute persistent MSK pain presentations. This report describes repeated emergency presentations of an individual with acute persistent MSK pain in their twenties living with mental health. Their mental health diagnoses included depression, mood disorders, and anorexia nervosa. This person also had mental health related inpatient admissions that were not captured under the retrospective record review for a large district hospital emergency department using the Systematized Nomenclature of Medicine Clinical Terms (SNOMED CT) classification system. This case report attempts to demonstrate that improving the understanding of preexisting vulnerabilities and mental health diagnoses may assist with informing healthcare design to develop specialised care pathways for acute injury presentations within triage settings.

## 1 BACKGROUND

Acute MSK pain represents a common cause for seeking emergent health care,^1^, ^2^ ^,^ ^3^ While the majority of acutely injured people should expect to recover spontaneously, 50% will transition from acute to chronic pain and disability.^4^, ^5^Data from robust clinical trials^6^ and prevalence studies have enabled the identification of those at risk of delayed recovery from MSK injury. However, interventions targeting known risk factors have yielded at best, only modest effects.^7^, ^8^ Furthermore, the most recent output from the global burden of disease in 2019 suggest MSK conditions are a leading cause of civilian years lived with disability. Alarmingly, this has not changed since 1990, suggesting research has had little effect on reducing the burdens of acute and chronic MSK conditions. Perhaps critical to this long‐standing problem is that research has not generated new mechanistic knowledge into why some people recover and others do not following their acute MSK presentation.^9^

Acute MSK pain also accounts for a large number of Emergency Department (ED) presentations, particularly in those patients considered to be frequent users.^10^ Multiple studies have investigated which factors are associated with frequent ED use, with a preexisting mental illness being one of the more commonly identified predictors.^11^ Despite this, few studies have investigated the mechanistic role preexisting mental illness plays in the manifestation of MSK pain. Perhaps reconceptualising the acute MSK injury (be it a slip‐and‐fall, motor vehicle collision, fracture, muscle strain affecting the spine or extremities) as both a stress‐ and tissue‐based injury is required to integrate and consider how preexisting diatheses such as mental health disorders^12^ influence the process of recovery. By considering existing diatheses, appropriate early screening tools and preventative treatments could be offered to improve clinical outcomes and avoid harmful secondary effects, such as opioid dependency, stigma, poor return to work outcomes, withdrawal from valued life roles, long‐term reliance on ineffective and costly management options as well as reducing ED encounters.

The association between mental illness and poor physical health is well established, with multiple studies reporting reduced life expectancy and higher number of chronic physical conditions in individuals with mental health disorders.^13^ For example, people living with mental health conditions tend to experience adverse physical health outcomes and significantly more medical conditions as compared to others without a history of mental health disorders. This is not to suggest the presence of a mental health disorder(s) predisposes a person to a life of chronic pain following adulthood injury requiring emergent care. While it is acknowledged that the ED environment presents a challenge, if not a trigger, to both busy, time‐strapped, clinicians, and the patients themselves, knowledge of preexisting diatheses could inform and streamline new clinical pathways for acute MSK injury on a patient‐by‐patient basis.

The case presented is that of a Caucasian woman in her twenties who presented to an ED multiple times (38 visits) over a 7‐year period (28 of which were in the span of one calendar year), and used to highlight the challenges for both the patient and the healthcare providers as well the opportunities for improving models of care for this type of patient.

## 2 CASE PRESENTATION

The repeated voluntary ED presentations (38 visits over 7 years, from January 2015–February 2021, though noted 28 presentations occurred within one calendar year, from January 2015–December 2015) were observed to be for persistent musculoskeletal (MSK) pain, involving an extremity and upper limb pain. Preexisting diagnoses of mood disorder, depression, and an eating disorder (anorexia nervosa) were also recorded at each ED presentation. This number of ED presentations over such an extended time period has been classified as persistent or ongoing frequent ED use in previous studies, a pattern of service usage which causes a significant burden for both the healthcare system and, notably, the person.^2^ It appears the individual was initially seeking support however may not have felt that the support and care needs were being met despite the repeated presentations. This retrospective record review was approved by the Northern Sydney Local Health District Human Research Ethics Committee, ethics approval number – 2021/STE02301: SSA.

## 3 DESIGN

A retrospective review of electronic medical records from January 2015 until July 2021 for ED presentations at a large urban district hospital serving a catchment of over 1.5 million people was performed. Data for acute presentations of MSK pain as well as mental health admissions over the preceding 12‐month periods was obtained.

### 3.1 Subject

This case report presents an individual in their twenties, with multiple ED presentations (38 visits) over a 7 year period (2015–2021). It is noted that 10 of these 38 visits spanned a 6‐year period whereas 28 visits occurred in 2015 alone. The reason for each presentation was classified by the treating ED physician/clinicians using the SNOMED CT system, a standardised, multilingual vocabulary of clinical terminology containing more than 300,000 medical concepts used by health care providers within the electronic exchange of clinical health information.^14^, ^15^ The SNOMED CT is made up of the numerical codes, known as concepts, used to identifying clinical information. The number of concepts used are largely if not completely dependent on the clinical setting and patient census. In this case, the number of concepts available in a busy urban ED with level 1 trauma status is in the thousands. The concepts are divided into different groups such as body structure, clinical findings, geography, location and biological products represented by different concepts based on the complexity of the presenting condition. The terminology classifies presentations under findings, disorders, diagnoses and similar with individual numbers. SNOMED CT classifies “findings” as observations which may be objective or subjective information from a primary source, including human observation whereas the term “disorder” refers to as an abnormal clinical state and are classified under the hierarchy of disease.^14^ SNOMED CT however also tends to be subjective and have the same description while referring to different concepts due to the ambiguity dependant on the triage.^16^

The ED admission data captured the date, the patient's reason for the visit to ED, MSK diagnosis provided at triage, and the preexisting MH diagnosis. Under SNOMED CT, findings refer to observations that exist at the time of recording, while disorder suggests an abnormal and underlying psycho‐physical pathological process that remains a vulnerability even post completion of treatment.^15^ For this case report we extracted only the ED presentations relating MSK/acute pain, the majority of which related to persistent MSK pain involving an extremity, as summarised in Table 1.

**TABLE 1:** Acute MSK pain presentations from January 2015.

No	Date	Reason For visit	MSK Diagnosis given at triage	MH diagnosis
1.	x Jan 2015	Pain, UL	Traumatic Dx of shoulder R (d)	UM[a]D
2.	x Jan 2015	MH—BD	Dx of shoulder J (d)	UM[a]D
3.	x Jan 2015	Pain, UL/shoulder	Dx of shoulder J (d)	UM[a]D
4.	x Jan 2015	Injury—Dx	Traumatic Dx of shoulder R (d)	UM[a]D
5.	x Jan 2015	Dx	Traumatic Dx of shoulder R (d)	UM[a]D
6.	x Jan 2015	Pain, UL/shoulder	Dx of shoulder J (d)	UM[a]D
7	x Feb 2015	MH—self harm	Open wound in ft (+ tendon inv.) (d)	UM[a]D
8.	x Mar 2015	MH—self harm	Multiple lacerations (d)	UM[a]D
9.	x Apr 2015	Injury—UL	Traumatic Dx of shoulder R (d)	Dysthymia; PTSD; DC
10.	x May 2015	Injury—UL	Dx of shoulder J (d)	Dysthymia; PTSD; DC
11.	x May 2015	Pain, UL/Shoulder	Dx of shoulder J (d)	Dysthymia; PTSD; DC
12.	x May 2015	Injury—Dx	Traumatic Dx of shoulder R (d)	Dysthymia; PTSD; DC
13.	x May 2015	Pain, UL/shoulder	Shoulder pain (f)	Dysthymia; PTSD; DC
14.	x May 2015	Pain, UL/shoulder	Shoulder pain (f)	Dysthymia; PTSD; DC
15.	x May 2015	Pain, UL/shoulder	Traumatic Dx of shoulder R (d)	Dysthymia; PTSD; DC
16.	x May 2015	Pain, J	Shoulder pain (f)	Dysthymia; PTSD; DC
17.	x Jun 2015	Injury—Dx	Traumatic Dx of shoulder R (d)	Dysthymia; PTSD; DC
18.	x Jun 2015	Injury—Dx	Pain in limb (f)	Dysthymia; PTSD; DC
19.	x Jun 2015	Pain, UL/shoulder	Shoulder pain (f)	Dysthymia; PTSD; DC
20.	x Jul 2015	Injury—Dx	Dx of shoulder J (d)	Dysthymia; PTSD; DC
21.	x Jul 2015	Pain, UL/shoulder	Traumatic Dx of shoulder R (d)	Dysthymia; PTSD; DC
22.	x Jul 2015	Pain, UL/shoulder	Dx of shoulder J (d)	Dysthymia; PTSD; DC
23.	x Jul 2015	Pain, J	Traumatic Dx of shoulder R (d)	Dysthymia; PTSD; DC
24.	x Aug 2015	Injury—Dx	Dx of shoulder J (d)	Dysthymia; PTSD; DC
25.	x Sep 2015	Pain, UL/Shoulder	Shoulder pain (f)	Anorexia nervosa (d)
26.	x Oct 2015	Pain, UL/Shoulder	Shoulder pain (f)	Anorexia nervosa (d)
27.	x Nov 2015	Injury—Dx	Dx of shoulder J (d)	Anorexia nervosa (d)
28.	x Dec 2015	Injury—UL	Traumatic Dx of shoulder R (d)	Anorexia nervosa (d)
29.	x Jan 2016	Pain, UL/shoulder	Dx of shoulder J (d)	anorexia nervosa (d)
30.	x Feb 2016	Injury— Dx	Dx of shoulder J (d)	Anorexia nervosa (d)
31.	x Jun 2016	Injury—UL	Dx of shoulder J (d)	Anorexia nervosa (d)
32.	x Aug 2016	Injury—UL	Closed Fx of phalanx of FNGR (d)	Anorexia nervosa (d)
33.	x Oct 2016	Injury—UL	Traumatic Dx of shoulder R (d)	Anorexia nervosa (d)
34.	x Mar 2017	Injury—UL	Dx of shoulder J (d)	Anorexia nervosa (d)
35.	x Sep 2017	Injury—UL	Dx of shoulder J (d)	Anorexia nervosa (d)
36.	x May 2020	Pain, UL/Shoulder	Shoulder pain (f)	Anorexia nervosa (d)
37.	x Oct 2020	Pain, UL/Shoulder	Dx of shoulder J (d)	Anorexia nervosa (d)
38.	x Feb 2021	Injury—Dx	Dx of shoulder J (d)	Anorexia nervosa (d)

Abbreviations: BD, behavioral disturbance; d, disorder; DC, dissociative convulsions; Dx, dislocation; f, finding; ft, foot; FNGR, Finger; Fx, fracture; J, Joint; MH, mental Health; MSK, musculoskeletal; +tendon inv., with tendon involvement; PTSD, post‐traumatic stress disorder; R, Region; UL, upper limb; UM[a]D, unspecified mood [affective] disorder.

*Note*: All the “x” are separate dates recording ED presentation/s on different days.

As summarised in Table 1, the repeated MSK/ acute pain related presentations observed over the 7‐year period were for persistent MSK pain involving an extremity.^15^ There were multiple mental health related admissions separate to the acute MSK pain presentation at ED over this period recorded initially for an unspecified mood (affective) disorder, progressing to dysthymia/ mood disorder, followed by a separate admission for post‐traumatic stress disorder (PTSD), a further mental health admission for dissociative convulsions, and the last captured admission was for anorexia nervosa (classified under eating disorder/s). This range of diagnoses also indicates the complexity of the patient's underlying mental health concerns, as well as a possible difficulty in identifying the most appropriate diagnosis and treatment.

Information regarding social circumstances, such as living independently or in supported accommodation, employment or education status, social supports both informal and informal including community mental health services, general practitioner and any nongovernmental organisations involvement was not available. Further the ED for this particular hospital within the health district is located within an area with a fairly low level of perceived socioeconomic disadvantage and close to the central business district, however, does cover areas of social housing for vulnerable populations.

## 4 DISCUSSION

This case study was produced following a retrospective record review of over 40,000 emergent care presentations. Here, we report on a specific patient with a long‐standing history of mental illness presenting to emergent care for a recurring isolated disorder of the MSK system over a 7 year period. Priority was given to assessing and managing the recurring MSK presentations. However, the same provision was not given to preexisting mental illness and their potential to contribute to, if not drive the, MSK issues. While the influence of mental health disorders in chronic pain have been well documented, there remains limited information for how these factors are collected and considered during an emergent care encounter for an MSK presentation. It remains plausible that such factors have potentially contributed to this patient's experience in seeking care for their MSK injury/disorder, and the association between preexisting mental health disorders and frequent ED presentations is well documented.^11^,^17^ It is noted that 28 presentations occurred every two‐weeks in 2015 alone whereas the remaining 10 (for the same persistent condition) occurred over the following 6 years. While it is not plausible to definitively state the precise reasons behind the number of visits for her shoulder pain in 2015 alone, one can only posit that “we” have yet to identify and use accessible, affordable, and reasonable objective markers for a patient's pain and ‘we’ have not yet reconciled these markers must be linked with the person's subjective report of their own experience. As Margot McCaffery (RN) stated, *Pain is whatever the experiencing person says it is, existing whenever and wherever the experiencing person says it does*.^18^

If “we” are to believe McCaffery, then it is imperative we recognise the person experiencing/reporting the pain is the closest thing to a “gold‐standard” of a pain biomarker available to “us”. This also fits with the words from Harvard psychologist, Daniel Gilbert, in his book *Stumbling on Happiness*, who says:

*“If we want to know how a person feels, we must begin by acknowledging the fact that there is one and only one observer stationed at the critical point of view. She may not always remember what she felt before, and she may not always be aware of what she is feeling right now. We may be puzzled by her reports, sceptical of her memory, and worried about her ability to use language as we do. But when all our hand wringing is over, we must admit that she is the only person who has even the slightest chance of describing ‘the view from in here’, which is why her claims serve as the gold standard against which all other measures are measured*.^19^

The available data details a total of 38 encounters for persistent MSK‐related pain or injury over a 7 year period ending in Feb 2021, classifying the patient as a *frequent‐flyer* of the ED.^20^ Upper limb pain and persistent MSK pain involving an extremity were the main reasons for ED presentation. However, no further mental health presentations for the ED or specialist care were identified which may be due to the type of data captured (or not) in the retrospective records. This data has been captured in the table attached with the case study illustrating the different ED presentations over the 7 year period (noting 28 in 2015).

The final mental health diagnosis provided in April 2017 reports a first‐time diagnosis of anorexia nervosa. It is important to note that up to 40% of people diagnosed with anorexia nervosa have comorbid personality disorders across the Cluster B traits, which are defined by the DSM as including impulsive behavioural patterns along with compulsive traits.^21^ Further, eating disorders are associated with a range of physical complications that may impact on repeated MSK presentations.^22^ Existing evidence suggests that pain is implicated in higher rates of generalised anxiety disorder (GAD), PTSD, substance misuse, and other comorbid disorders^23^ resulting in a further reduction in functionality, recalcitrant treatment response and increased health care costs. It appears that people living with mental health conditions receiving inadequate treatment remain at risk of experiencing other comorbid health conditions and it remains plausible that the repeated acute pain/MSK presentations observed may be due to an unrecognised eating disorder phenotype. An improved understanding of preexisting vulnerabilities/resiliencies associated with repeated acute care presentations and triage processes may inform healthcare redesign to streamline more bespoke care pathways for people with acute MSK injury.

People living with a mental illness can experience concomitant physical symptoms, which can in turn result in a shortened life span, increased comorbidities, and a lowering in their quality of life due to a mix of disparity in healthcare access and utilisation.^24^,^17^ Unfortunately, these physical symptoms have been largely attributed to underlying psychiatric conditions.^25^ It is possible that living with a mental illness may lead to a delay in establishing the correct diagnosis and intervention required to adequately address the physical and mental signs/symptoms, which could result in an inappropriate plan of care on discharge from the ED.

Early onset mental health disorders have shown to increase risk for lifelong adversity,^26^ contributing to health inequity. There are several studies on the observations of, and reporting on, an increasing number of young people presenting to the ED with mental health disorders.^27^ The intensely stimulating environment of the ED may prove a therapeutic challenge for an acutely injured person with or without history of previous mental health disorders. The trauma, distress, pain, and expectations around recovery are complex for people living with a mental illness. Adding to this complexity are preexisting stress, pain, mental ill‐health, and early life adversity as all could influence the clinical course on a patient‐by‐patient basis following acute injury. Such complexity can, and likely does, place further demands on the resources within an ED workforce and resources.^28^

### 4.1 Limitations

The retrospective data collection only captured ED presentations for acute MSK pain at a single ED location, and therefore additional presentations at other EDs, as well as presentations for reasons other than MSK pain were omitted from the dataset. Additional information such as any triggers prior to presentation and existing supports and services the patient may have engaged with in the community were also not available. Given this case report was based on the retrospective study of de‐identified data, it was also not possible to provide information regarding the patient's perspective.

This case highlights the need to explore, build, develop, and embed new pathways to realise informed transitions of care for patients through the linkage of electronic medical records. The ED and outpatient clinics are two examples where preexisting diagnoses and treatment for acute care presentations are poorly coded, and multiple repeated care episodes are frequent. There remains a very wide range of clinically relevant research questions that would be enhanced by linked data. The medium and long term outcomes of all care types and variations would be usefully examined, and linked data might also provide much richer information on risk factors and social determinants of health outcomes.

While such infrastructure build‐out would require a skilled workforce, ethical and governance review, and a prospective patient consent system, it could become an enduring source of real‐world evidence about transitions of care—allowing clinicians within healthcare service organisations, such as local health districts (LHDs), to partner with academics on research projects that help them to better understand their patients outside of direct care, including what happens to them before and after they are discharged. The opportunity to predict long‐term health outcomes of patients, would empower the delivery of better targeted care plans based on the awareness of preexisting diagnoses that could influence the patient's experience with a ‘later‐in‐life’ injury, disease, disorder

### 4.2 Learning points/take home messages

- Future work should aim to refine chart review data towards identifying any patterns observed within specific groups and explore interventions offered and the relative efficacy of these care pathways between people living with a significant mental disorder/ illness as compared to others. It is also important to explore unconscious bias displayed by the clinicians while documenting the intensity of pain in the presence of mental ill health and the capacity of the person presenting at an emergency setting to offer appropriate interventions and discharge care pathways.
- We raise this case to stimulate awareness and encourage multidisciplinary discussion that reconceptualising acute MSK injury event as both potentially injurious and distressing, influenced by preexisting vulnerabilities or resiliencies of the person and the socioenvironmental context within which the person lives and functions.
- Preexisting psychopathology may be a risk‐factor for poor recovery from an MSK presentation. Previous studies have shown as association between mental illness and multiple ED presentations, although the effect of unrecognised mental health diagnoses has yet to be investigated. This single‐study case followed the repeated ED admissions experienced by a young female person with preexisting mental health diagnoses for acute MSK concerns and highlighted the need to explored individualised treatment pathways for patients with similar presentations.

## AUTHOR CONTRIBUTIONS

**Priya Arora:** Conceptualisation; data curation; formal analysis; funding acquisition; investigation; methodology; project administration; resources; validation; visualisation; writing – original draft; writing – review and editing. **James M Elliott:** Conceptualisation; formal analysis; funding acquisition; methodology; project administration; resources; supervision; validation; writing – review and editing. **Fereshteh Pourkazemi:** Conceptualisation; data curation; formal analysis; methodology; supervision; writing – review and editing. **Roxanna Nasseri Pebdani:** Methodology; supervision; writing – review and editing.

## FUNDING INFORMATION

Private funding.

## ETHICS STATEMENT

Ethical approval to report this case was obtained from the Northern Sydney Local Health District (NSLHD) Human Research Ethics Committee (HREC), ethics approval number—2021/STE02301: SSA.

## CONSENT

Written informed consent was obtained from a legally authorized representative(s)—NSLHD HREC for anonymized patient information to be published in this article. As this research is conducted under the ethics approval, a consent waiver is requested as the dataset provided is non‐identifiable. As this case falls under an approved retrospective study interrogating our electronic medical record to determine the number of emergency medicine presentations for musculoskeletal injuries in people with a recorded history of diagnosable psychopathology. This case falls under an approved retrospective study interrogating our electronic medical record to determine the number of emergency medicine presentations for musculoskeletal (MSK) injuries in people with a recorded history of diagnosable psychopathology. This retrospective body of work obtained over 44,000 records of de‐identified data covering seven years. The identity of any patient in this dataset remains unknown to us and it is and was not possible for us to contact them to provide consent. When querying the available database, we were surprised by this particular case detailing a large number of presentations for MSK pathologies and a known history of diagnosable psychopathology.

## Data Availability

Data available on request due to privacy/ethical restrictionsThe data that support the findings of this study are available on request from the corresponding author. The data are not publicly available due to privacy or ethical restrictions.
